# Complement C3 is associated with cancer-induced frailty: genetic and clinical evidence

**DOI:** 10.3389/fnut.2026.1953476

**Published:** 2026-09-30

**Authors:** Chaobao Zhang, Ting Zhao, Weiyi Li, Zhan Shi, Zhijun Bao

**Affiliations:** 1 Shanghai Key Laboratory of Clinical Geriatric Medicine, Shanghai Institute of Geriatrics and Gerontology, Huadong Hospital, Shanghai Medical College, Fudan University, Shanghai, China; 2 National Clinical Research Center for Aging and Medicine, Huashan Hospital, Fudan University, Shanghai, China; 3 Department of Pulmonary and Critical Care Medicine, Huadong Hospital, Shanghai Medical College, Fudan University, Shanghai, China; 4 Department of Oncology, Huadong Hospital, Shanghai Medical College, Fudan University, Shanghai, China

**Keywords:** cachexia, cancer, component C3, frailty, PheWAS

## Abstract

**Background:**

Frailty is a degenerative geriatric syndrome characterized by declining muscle mass and function, unintentional weight loss, and fatigue. It is a key risk factor for many geriatric diseases and poor postoperative outcomes. Currently, pathogenesis remains unclear, and apart from nutritional support and exercise, no pharmacological interventions are available. Notably, the frailty phenotype is also a major feature in patients with advanced-stage cancer, and both frailty and advanced cancer are characterized by dysregulation of nutritional and energy metabolism. Consequently, cancer serves as a novel accelerated model for investigating the mechanisms underlying frailty development.

**Methods:**

The phenome-wide association study (PheWAS) framework was employed to systematically analyze the causal relationship between 264 cancer types and frailty, and to identify candidate cancer types directly causally associated with frailty. A large-scale analysis was conducted to assess the causal relationship between “candidate cancer- Icelandic peripheral blood proteome-frailty” and to further explore the characteristics and patterns of the key proteins mediating cancer-induced frailty using the two-sample Mendelian randomization method. Finally, the correlation between key candidate proteins and frailty was verified using peripheral blood from a 100-case colorectal cancer (CRC) cohort.

**Results:**

The PheWAS analysis identified 21 cancer types with significant causal associations with frailty among the 264 cancers examined. An in-depth analysis of the top 5 cancer types revealed that the mechanisms by which they induce frailty exhibit both prominent shared features and distinct characteristics. Shared signaling pathways included stress responses, immune processes, and metabolic pathways. Among these, immune-related pathways involving complement component C3 (C3), lipocalin-2 (LCN2), and coagulation factor V (F5) emerged as key shared mechanisms across multiple cancer types. Finally, validation in an independent clinical cohort of patients with CRC (*n* = 100) demonstrated that circulating C3 levels were positively correlated with frailty severity and the frailty-related comorbidity index, and negatively correlated with serum albumin levels.

**Conclusion:**

This study systematically elucidated the causal relationships between cancer and frailty, identified multiple candidate proteins that may drive frailty progression, and demonstrated that complement C3 is associated with frailty development. Furthermore, we propose that cancer serves as a novel accelerated model for investigating the mechanisms underlying frailty. These findings provide new insights into the biological mechanisms underlying frailty and identify a series of promising molecular targets for future mechanistic studies and therapeutic interventions.

## Introduction

Frailty is a common age-related degenerative syndrome, with a prevalence of approximately 10–20% among individuals aged 60 years and older (1). Frailty is also a major risk factor for multiple age-associated diseases and adverse postoperative outcomes (2). It has become an essential component of preoperative risk assessment for older adults undergoing complex surgical procedures. A wide range of conditions, including diabetes mellitus, infectious stress, depression, and kidney disease, have been identified as risk factors for frailty. Consequently, the frailty index, primarily reflecting the cumulative comorbidity burden, is widely used in clinical practice to assess frailty (3). Clinically, frail individuals typically present with fatigue, weakness, slowness, low physical activity, and unintentional weight loss, constituting the core components of commonly used frailty screening and assessment tools (4). Although emerging evidence suggests that immune senescence (5), inflammatory microenvironment alterations (6), mast cell activation (7), accelerated aging, and metabolic dysfunction contribute to frailty development and progression, the underlying molecular mechanisms remain poorly understood. As a complex clinical syndrome, frailty lacks robust *in vitro* cellular models, which has substantially hindered therapeutic agent development. Currently, no pharmacological treatment has been approved for frailty. The current management of frailty primarily relies on nutritional support, physical exercise, and comprehensive health management to alleviate symptoms or delay disease progression (8).

The limited understanding of the molecular mechanisms underlying frailty, together with the lack of appropriate *in vitro* cellular models, has hampered the efficiency of the conventional translational research paradigm, aiming to translate basic research findings into disease mechanisms and therapeutic targets for clinical applications. As a geriatric syndrome rather than a single disease entity, a reverse translational research paradigm, from clinical observations to basic mechanistic investigations, may provide a more effective strategy for elucidating frailty pathogenesis and identifying potential therapeutic interventions. Cancer, one of the most prevalent diseases in older adults, frequently induces a cachectic state characterized by rapid weight loss, skeletal muscle wasting, impaired muscle function, and fatigue, closely resembling the clinical manifestations of frailty (9, 10). Cancer cachexia affects up to 80% of patients with advanced malignancies and directly accounts for approximately 20–40% of cancer-related deaths (11). Similar to frailty, no effective pharmacological therapy is currently available for cancer cachexia. Clinical management mainly consists of nutritional support and exercise interventions (9), whereas therapies targeting growth differentiation factor 15 (GDF15) are still under clinical evaluation. Recent studies on cancer cachexia have identified several circulating factors, including GDF15 and insulin-like growth factor-binding protein 1, that directly promote skeletal muscle atrophy (12–14). Notably, circulating levels of these factors increase with age (15), suggesting that they may possess frailty-promoting potential. These observations indicate that cancer cachexia and frailty may share common pathophysiological mechanisms. Consequently, cancer serves as an accelerated model of frailty, providing an efficient platform for investigating the molecular mechanisms underlying frailty and developing potential intervention strategies.

Recently, Mendelian randomization (MR) has emerged as a powerful approach for inferring causal relationships between exposures and outcomes (16). Using genetic variants as instrumental variables (IVs), MR leverages the random assortment of alleles during meiosis to minimize confounding and reverse causality that commonly affect observational epidemiological studies. Genome-wide association studies (GWASs) have identified thousands of genetic variants associated with a broad spectrum of complex diseases, circulating proteins, metabolites, and other molecular traits (17, 18). These advances have made it possible to move beyond conventional association-based studies by leveraging MR, a causal inference approach, to efficiently investigate the causal relationships between multiple diseases and frailty on a large scale, thereby facilitating the identification of key mechanisms underlying the development of frailty.

In this study, we have adopted a phenome-wide strategy combined with large-scale MR analyses to systematically investigate the causal relationships between 264 cancer types and frailty. Furthermore, a three-step MR framework integrating cancer-circulating plasma proteome-frailty has been employed to identify the key proteins mediating cancer-induced frailty and to characterize their molecular functions and shared biological patterns. Finally, the associations between the identified candidate proteins and frailty have been validated using peripheral blood samples from an independent cohort of 100 patients with colorectal cancer (CRC).

## Materials and methods

### Core implementation strategy

Given the methodological advantage of MR in enabling causal inference while minimizing confounding, we employed a large-scale two-sample MR framework to systematically investigate causal relationships between diseases and circulating proteins. Following the analytical framework of cancer diseases → circulating blood proteome → frailty, we aimed to identify the key protein drivers underlying cancer-associated frailty. Specifically, we first conducted a large-scale two-sample MR phenome-wide association study to evaluate the causal effects of 264 cancer types on frailty, thereby identifying candidate cancers with significant causal associations. Next, we performed two-sample MR analyses to identify candidate circulating perturbation factors (P1) using the candidate cancers as exposures and the circulating blood proteome as the outcome. Subsequently, we treated the circulating proteome as the exposure and frailty as the outcome in a large-scale two-sample MR analysis to identify candidate protein drivers of frailty (P2). The intersection of the candidate circulating P1 and the candidate P2 was then defined as the key molecular drivers mediating the causal effect of cancer on frailty. Finally, the associations of selected key drivers were validated using blood samples from a clinical cohort.

### GWAS data acquisition

The GWAS data for neoplastic disease were obtained from FinnGenResearch’s data (version 12) released in May 2025^1^. Detailed information can be found in the Supplementary Tables S1, S2. Frailty phenotype GWAS data were sourced from a recent large-scale GWAS, encompassing 386,565 participants of European descent registered in the UK Biobank (UKB) (19). The frailty phenotype was assessed using five criteria: Low physical activity, slow walking speed, weight loss, exhaustion, and weak grip strength. Plasma proteomic data were obtained from deCODE Genetics (deCODE), reporting a GWAS of plasma protein levels measured with 4,907 aptamers in a cohort of 35,559 Icelanders (20). Given that the original studies for the exposure and outcome data used in this MR study were conducted in different countries, there is likely no overlap in the populations.

### Clinical cohort validation

The clinical validation cohort of patients with CRC was recruited from the Department of Oncology at Huadong Hospital, Affiliated with Fudan University. Consecutive patients were enrolled between February and July 2026, following approval by the institutional ethics committee (Ethics Approval No. 2025K334). CRC diagnosis was established based on histopathological examination of colonoscopic biopsy specimens or surgically resected tissues, along with morphological and immunohistochemical evaluation. All diagnoses were independently confirmed by at least two experienced pathologists. Tumor stage was determined according to the American Joint Committee on Cancer Tumor-Node-Metastasis (TNM) classification system by evaluating the extent of primary T invasion, regional N involvement, and distant M. Patients were subsequently classified as stage I, II, III, or IV. The clinical characteristics were obtained from electronic medical records and supplemented with follow-up data. The baseline variables included sex, age at diagnosis, smoking history, body mass index, biochemical parameters (including serum albumin and complement C3 levels), comorbidity number, and the Age-adjusted Charlson Comorbidity Index (aCCI). The aCCI was calculated as the sum of the Charlson Comorbidity Index score and the corresponding age-adjustment score (21). Patients with severe edema, fever or active infection, severe immunodeficiency, autoimmune diseases, or who were unable to complete the questionnaire due to objective limitations were excluded. Frailty status was subsequently assessed using a standardized questionnaire, and only participants with complete questionnaire data were included in the final analysis. Frailty was evaluated using a questionnaire adapted from the National Health and Nutrition Examination Survey (NHANES) (22). The questionnaire was developed based on the Fried frailty phenotype and assessed four items: Exhaustion, weakness, low physical activity, and weight loss. This adapted instrument strictly follows the frailty phenotype proposed by Fried et al. (4) and was subsequently validated by Rockwood et al. (22). It has been widely used to assess frailty in NHANES-based research (23).

### Statistical analysis

#### Selection of IVs

Instrumental variables were as follows: Single-nucleotide polymorphisms (SNPs) were selected using genome-wide significance criteria (*p* < 5 × 10^−6^) and clumped for independence using linkage disequilibrium threshold R^2^ < 0.001 within a 10,000 kb window (24). SNPs associated with the outcome at the Bonferroni-corrected significance level (*p* < 0.05) were excluded. SNP effects were harmonized between the exposure and outcome data at the allele levels.

#### MR and statistical analysis

Cancer-related diseases, frailty, and 4,907 blood protein traits were alternately treated as exposures and outcomes to investigate potential causal relationships. MR analyses were performed using three complementary methods: (1) Inverse-variance weighted (IVW), (2) Weighted median (WM), and (3) MR-Egger regression. The IVW method was the primary analytical approach, whereas WM and MR-Egger methods were used to assess the robustness of the causal estimates (25). A series of sensitivity analyses was conducted to evaluate the reliability of MR results. Heterogeneity among IVs was assessed using Cochran’s Q statistic (26). When significant heterogeneity was detected (*p* < 0.05), a random-effects IVW model was applied; otherwise, a fixed-effects IVW model was used. Horizontal pleiotropy was evaluated using the MR-Egger regression intercept and the MR-PRESSO framework to identify potential outlier variants (27). Associations with evidence of horizontal pleiotropy (*p* < 0.05) were excluded from the final results. All statistical analyses were performed using TwoSampleMR and MR-PRESSO packages in R (version 4.3.1^2^). Forest plots and bubble plots were generated using the Forestploter and ggplot2 packages via the SangerBox online analysis platform^3^.

#### Clinical cohort statistical analysis

Clinical observational data (*N* = 100) were analyzed using appropriate R packages (version 4.3.1). Comparisons between two groups were performed using two-tailed Student’s *t*-tests, whereas comparisons among multiple groups were conducted using one- or two-way analysis of variance (ANOVA), as appropriate. Categorical variables were compared using the chi-square test. For multiple comparisons, *p*-values were adjusted using the Bonferroni correction. Given the relatively small magnitude of C3 values, C3 was rescaled by a factor of 10 such that the corresponding odds ratio represented the effect associated with a 0.1-unit increase in the original C3 value. Multivariable ordinal logistic regression based on the proportional odds model was performed to assess the independent association between C3 and frailty severity, adjusting for age, sex, age-adjusted Charlson Comorbidity Index (aCCI), body mass index (BMI), smoking status, serum albumin level, tumor stage, and comorbidity burden. Adjusted odds ratios (aORs) with 95% confidence intervals (CIs) and two-sided *p* values were reported. Statistical significance was consistently denoted in all figures and tables as follows: *p < 0.05, **P < 0.01, and ***p < 0.001.

## Results

### Eight percent of cancer types exhibit a direct causal relationship with frailty

We used GWASs for 264 cancer-related diseases, defined according to the International Classification of Diseases (ICD) coding system, as the exposure, and the GWAS of the frailty phenotype derived from the UKB as the outcome (Supplementary Table S1). A standard two-sample MR framework was applied to evaluate the causal relationship between cancer and frailty (Figure 1A), and the IVW estimate distribution was visualized using a volcano plot. The results revealed that 21 of the 264 cancer types exhibited a significant causal association with frailty, accounting for approximately 8% of all cancer types examined (Figure 1B). Forest plot analysis further demonstrated that the majority of these 21 cancer types exhibited positive causal effects on frailty, including several common cancers, such as CRC, breast cancer, hemangioma, lipomatous neoplasm, and kidney cancer (Figure 1C). These findings suggest that frailty associated with most cancers primarily arises through indirect mechanisms, whereas several common cancer types also exert substantial direct causal effects on frailty. Consequently, the five cancer types with sample sizes exceeding 500 and the strongest statistical evidence were selected for subsequent in-depth analyses.

**Figure 1:**
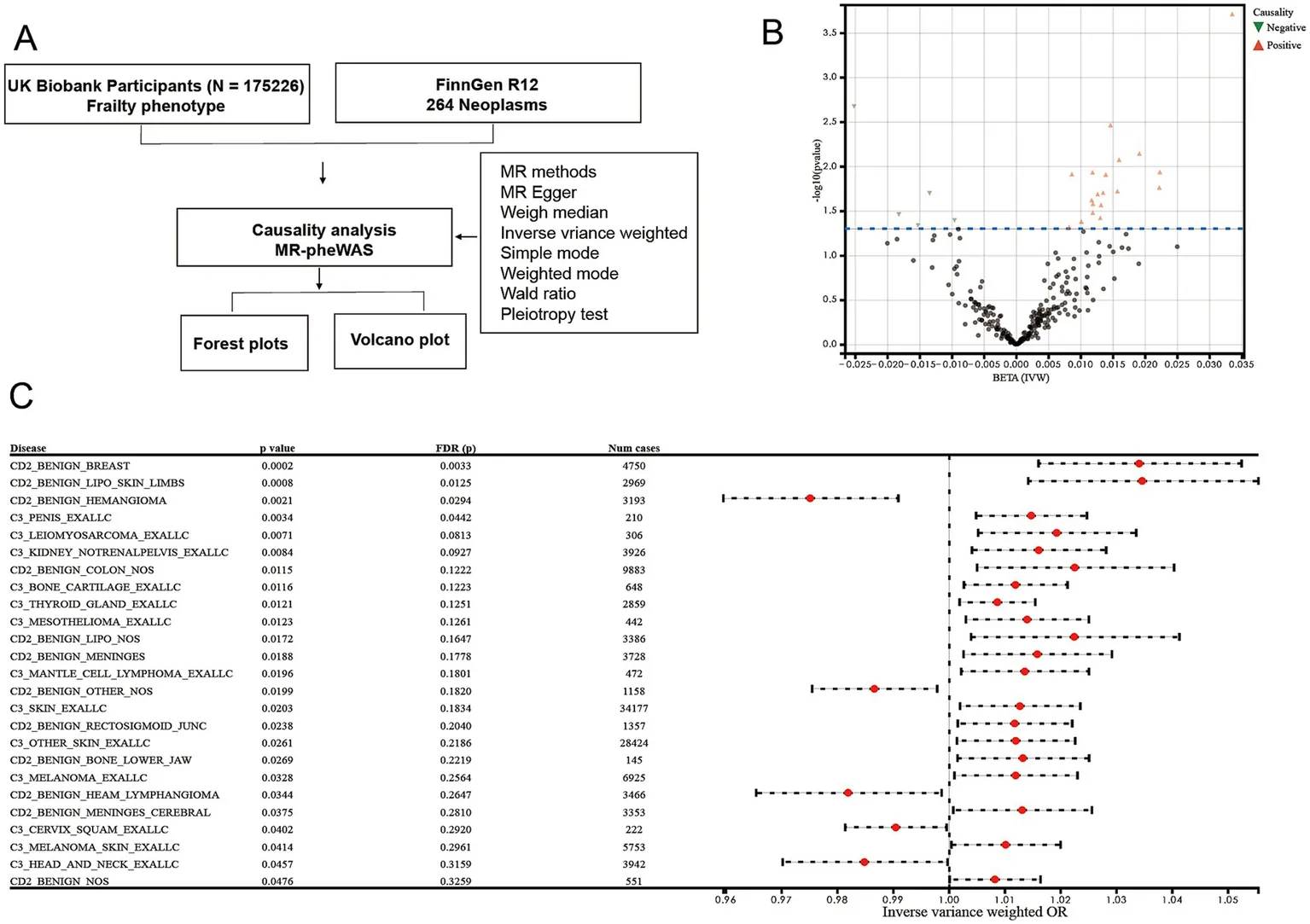
Causal effects of cancer-related diseases on frailty. **(A)** Analytical framework flowchart. **(B)** Volcano plots illustrating the MR analysis of the associations between cancer-related diseases of the Finnish cohort and the frailty phenotypes of the UKB cohort. **(C)** Forest plot exhibiting the causal effects of 21 distinct cancer-related diseases on frailty phenotypes identified through MR. IVW, inverse-variance weighted; OR, odds ratio.

### Distribution of mediating proteins underlying cancer-associated frailty across different cancer types

The GWASs of the five selected cancer types were used as exposures, while GWASs of 4,907 circulating plasma proteins from the Icelandic cohort were used as outcomes, to systematically investigate the causal effects of these cancers on the peripheral blood proteome using two-sample MR analysis (Supplementary Tables S2–S6). Simultaneously, we performed an additional large-scale MR analysis using GWASs of the 4,907 circulating proteins as exposures and the frailty GWAS as the outcome to identify proteins causally associated with frailty (Figure 2A and Supplementary Table S7). The volcano plot displays that 108 circulating proteins have a direct causal relationship with frailty (Figure 2B). Notably, the number of proteins causally influenced by different cancer types varied substantially (Figure 2B). Kidney cancer exhibited the largest number of causally associated circulating proteins, with up to 300 significant proteins identified. These findings indicate that different cancer types exert markedly distinct perturbations in the circulating proteome.

**Figure 2:**
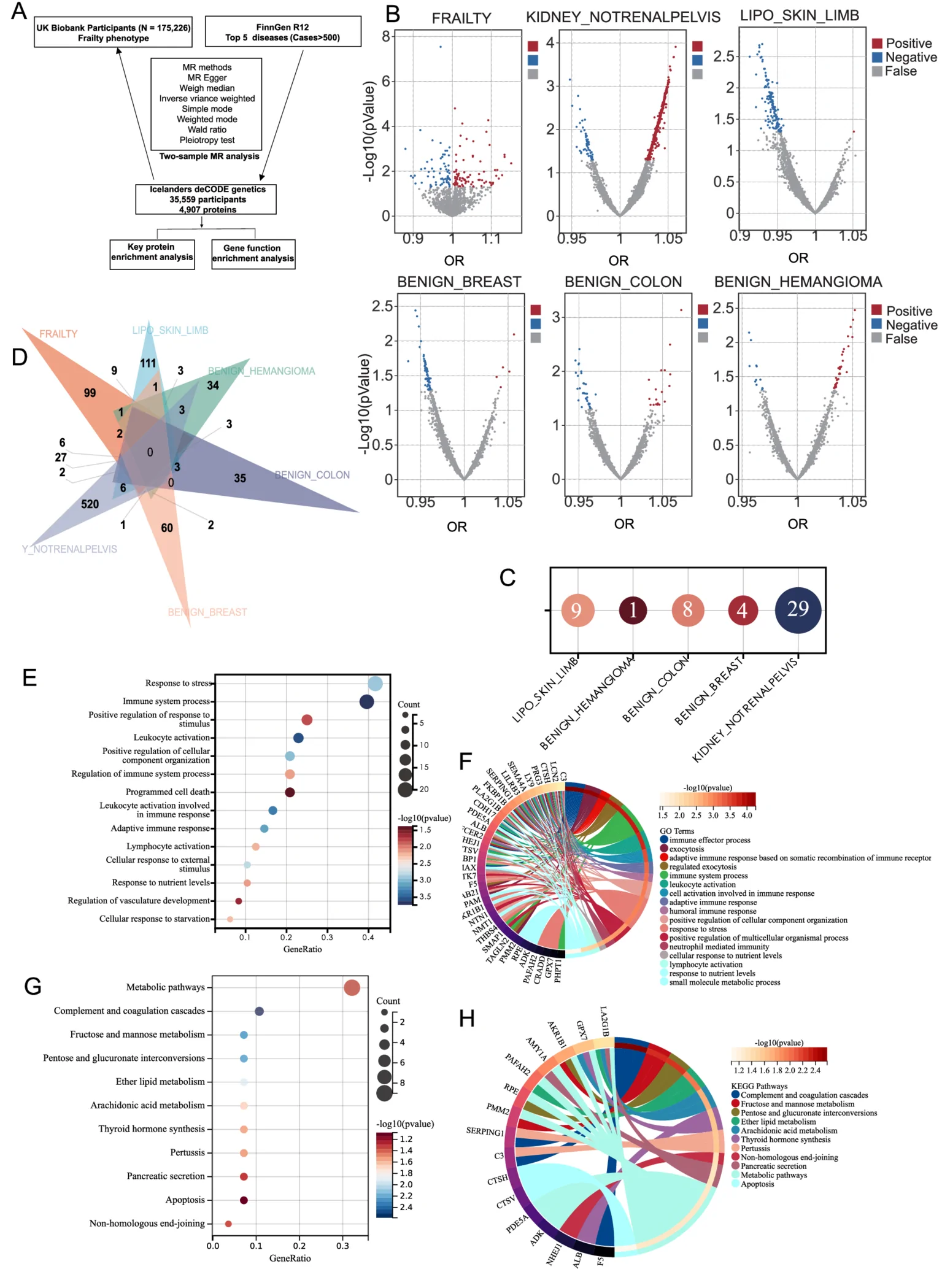
Screening of key blood protein mediators of the causal effect of cancer-related diseases on frailty. **(A)** Analytical framework flowchart. **(B)** Volcano plots illustrating the MR analysis on the associations between plasma proteins of the Icelander cohort and frailty or cancer-related diseases. **(C)** Heatmap of key protein numbers that drive frailty in different cancer-related diseases. **(D)** Venn diagram visualizing MR-identified protein overlap across five cancer-related diseases. **(E)** GO biological function analysis of key proteins driving frailty in cancer-related diseases. **(F)** GO chord diagram exhibiting the key proteins enriched in each biological functional item. **(G)** KEGG pathway enrichment analysis of key proteins driving frailty from cancer-related diseases. **(H)** KEGG chord diagram exhibiting the key proteins enriched in each pathway item.

To identify potential key mediators linking cancer to frailty, we intersected the MR-positive protein candidates identified for each cancer type with the proteins that exhibited causal effects on frailty. This approach yielded candidate mediating protein sets for each cancer type. The number of identified mediators differed considerably among cancers. For example, 29 mediating proteins were identified for kidney cancer, whereas only one mediating protein was identified for hemangioma (Figure 2C). Notably, there was minimal overlap among the mediating proteins identified across different cancer types, suggesting that distinct cancers promote frailty through largely independent molecular mechanisms (Figure 2D). Gene ontology (GO) enrichment analysis of all identified mediating proteins revealed significant enrichment in biological processes related to cellular stress response, immune regulation, and programmed cell death (Figure 2E). Notably, proteins such as C3, LCN2, and CTSH were enriched across multiple functional categories, including immune regulation and nutrient sensing, indicating that they may serve as central regulatory nodes linking cancer to frailty (Figure 2F). Kyoto encyclopedia of genes and genomes (KEGG) pathway enrichment analysis revealed significant enrichment in metabolic pathways, the complement cascade, and apoptosis-related signaling pathways (Figure 2G). Among the identified mediators, PLA2G1B, GPX7, and AKR1B1 participated in multiple enriched signaling pathways (Figure 2H). Collectively, these findings suggest that cancers primarily drive frailty through dysregulation of immune, metabolic, and nutrient-sensing pathways.

### Distinct and shared molecular pathways underlying cancer-associated frailty

To further elucidate the molecular mechanisms by which different cancers contribute to frailty, we performed pathway-level analysis. GO enrichment analysis was conducted on the blood proteins perturbed by each cancer type, and the enriched biological functions were compared with those of blood proteins associated with frailty (Supplementary Tables S8–S13). The results revealed substantial heterogeneity in the number of shared molecular functions across different cancer types. Specifically, kidney cancer shared 474 GO functional terms with frailty-associated proteins, whereas hemangioma shared only 145 terms (Figure 3A). These findings suggest that the molecular functions of proteins mediating cancer-associated frailty exhibit distinct characteristics specific to each cancer type.

**Figure 3:**
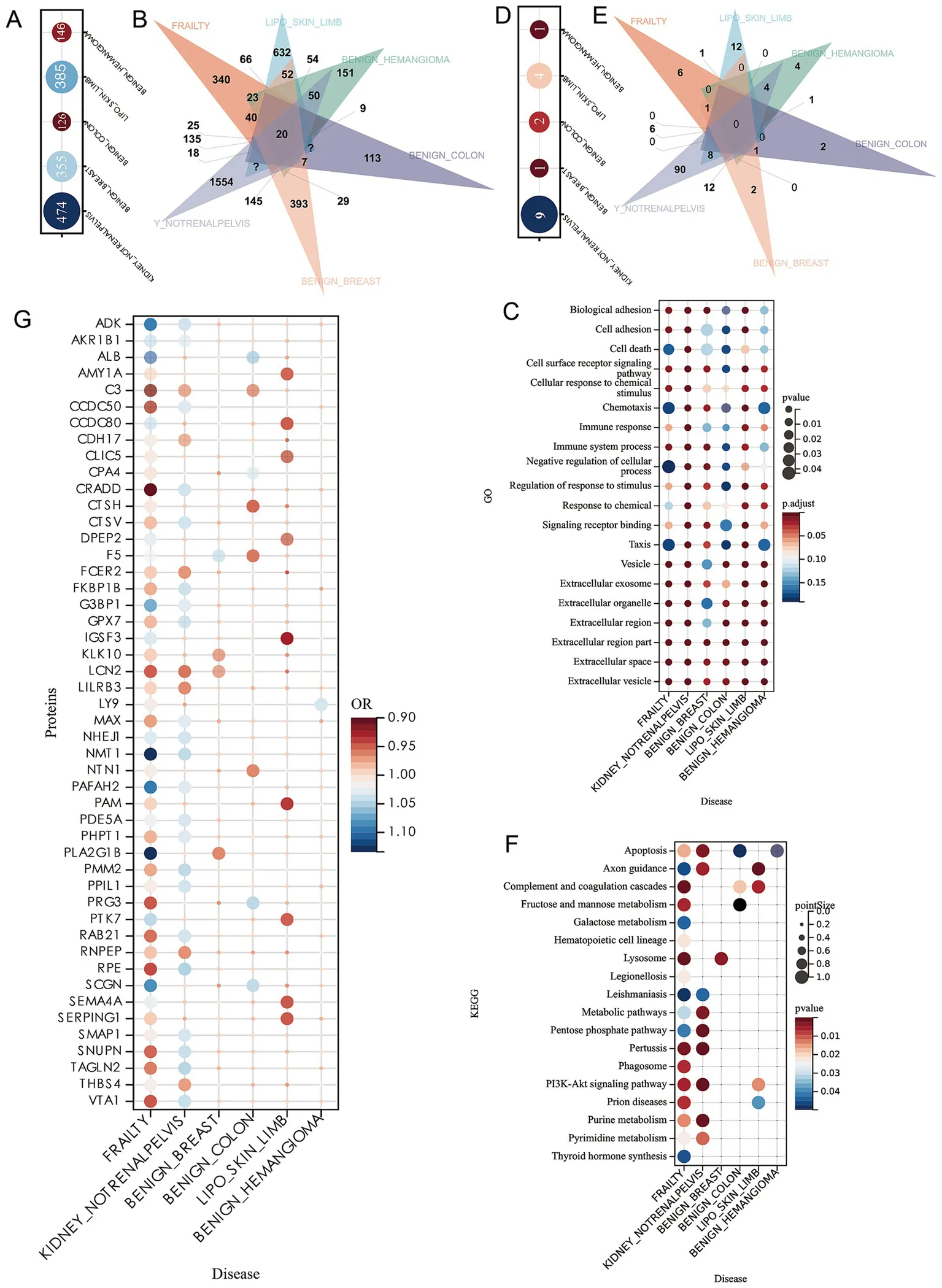
Common and distinct characteristics of key frailty-associated proteins across different cancer-related diseases. **(A)** Heatmap exhibiting differences in GO term number enriched by blood proteins shared between frailty and different cancer-related disease perturbations. **(B)** Venn diagram exhibiting GO term overlap enriched by blood proteins shared between frailty and different cancer-related disease perturbations. **(C)** Bubble plot exhibiting the common GO terms enriched by blood proteins shared between frailty and different cancer-related disease perturbations. **(D)** Heatmap exhibiting differences in KEGG pathway number enriched by blood proteins shared between frailty and different cancer-related disease perturbations. **(E)** Venn diagram exhibiting the KEGG pathway overlap enriched by blood proteins shared between frailty and different cancer-related disease perturbations. **(F)** Bubble plot exhibiting the common KEGG pathways and **(G)** shared and disease-specific characteristics of blood proteins shared between frailty and different cancer-related disease perturbations.

The intersection analysis of GO enrichment results from the 5 cancer types identified 20 common functional terms shared across all cancers (Figure 3B). These common biological functions were primarily associated with immune regulation, apoptosis, stress responses, and exosome-related processes, indicating that they represent conserved molecular mechanisms underlying cancer-associated frailty (Figure 3C). We further validated these shared and cancer-specific characteristics using KEGG pathway enrichment analysis (Supplementary Tables S14–S19). Kidney cancer shared the most KEGG pathways with frailty, whereas hemangioma shared the fewest (Figure 3D). Although no KEGG pathway was shared by all five cancer types (Figure 3E), several pathways, including apoptosis, complement signaling, and the PI3K signaling pathway, were enriched in three cancer types (Figure 3F). These findings further support the existence of both shared and cancer-specific molecular mechanisms through which different cancers contribute to frailty.

To further investigate the key mediators underlying these mechanisms, we compared the candidate mediating proteins identified in the five cancer types. None of the five cancers shared a common key protein. Among the 48 candidate mediator proteins identified, only three, C3, F5, and LCN2, were shared by two cancer types. This observation further suggests that the molecular mediators driving cancer-associated frailty comprise conserved and cancer-specific components.

### C3 concentration is positively associated with frailty severity in a CRC cohort

Through large-scale MR screening, we identified C3, F5, and LCN2 as three high-frequency key driver proteins of frailty across five cancer types. Given that previous studies have implicated members of the F5 family and LCN2 in cancer-associated frailty (28, 29), we selected C3 for further validation in an independent clinical cohort. A total of 100 patients with CRC were classified into robust, pre-frailty, and frailty groups according to the frailty phenotype criteria (Table 1). Peripheral blood C3 concentrations differed significantly among the three groups (Table 1), with circulating C3 levels increasing progressively with increasing frailty severity (Figure 4A). To further evaluate the clinical relevance of C3, the number of comorbidities was converted to a comorbidity index. Circulating C3 concentrations were positively correlated with the comorbidity index, indicating that elevated C3 levels were associated with a greater comorbidity burden (Figure 4B). Moreover, serum albumin, a well-established clinical indicator of frailty (30), was significantly negatively correlated with circulating C3 concentrations (Figure 4C). Notably, no significant association was observed between circulating C3 levels and tumor stage (Figure 4D). Interestingly, we performed a multivariable regression analysis adjusting for common covariates associated with frailty, including age, sex, comorbidities, serum albumin levels, smoking status, and tumor stage. The results showed that C3 remained independently associated with frailty severity after adjustment for these covariates (Table 1). Collectively, these findings demonstrate that circulating C3 concentrations are positively associated with frailty severity in patients with CRC, and that this association is independent of tumor stage.

**Table 1:** Baseline clinical characteristics and multivariable ordinal logistic regression analysis of frailty severity in the colorectal cancer cohort.

Characteristic	Total (*N* = 100)	Robust (NF) (*N* = 28)	Pre-frailty (PF) (*N* = 47)	Frailty (F) (*N* = 25)	Between groups			*p*-value	FDR(*P*)	Multivariable logistic regression	
					NF VS. PF (*P*)	NF VS. F (*P*)	PF VS. F (*P*)			OR (95% CI)	*P*-value
Female	56(56.00%)	18(64.29%)	27(57.44%)	11(44.00%)	0.7330	0.2283	0.4008	0.4876	1.0000	1.40 (0.56–3.56)	0.469
Age	64.86 ± 12.69	63.66 ± 13.01	58.96 ± 13.65	66.02 ± 11.19	0.0175	0.0058	0.2592	0.0076	0.0684	1.01 (0.96–1.06)	0.736
BMI	23.89 ± 3.088	24.46 ± 2.659	23.60 ± 3.314	23.80 ± 3.122	0.2426	0.2426	0.8003	0.497	1.0000	0.99 (0.83–1.17)	0.893
Smoking	46(46.00%)	14(50.00%)	20(42.56%)	12(48.00%)	0.6989	1.0000	0.8464	0.8004	1.0000	1.86 (0.72–5.01)	0.205
Albumin	28.81 ± 2.834	30.04 ± 2.835	28.68 ± 2.687	27.68 ± 2.673	0.0421	0.0031	0.1362	0.0083	0.0747	0.90 (0.72–1.10)	0.313
aCCI	6.060 ± 1.814	5.143 ± 1.779	6.043 ± 1.615	7.120 ± 1.691	0.0277	0.0001	0.0099	0.0002	**0.0018**	1.17 (0.83–1.69)	0.382
C3	1.165 ± 0.2262	0.9846 ± 0.1522	1.152 ± 0.1587	1.391 ± 0.2129	<0.0001	<0.0001	<0.0001	<0.0001	**0.0009**	2.19 (1.68–2.97)	**<0.001**
Comorbidity number	4.180 ± 1.749	3.357 ± 1.870	4.149 ± 1.668	5.160 ± 1.248	0.0613	0.0002	0.0097	0.0006	**0.0054**	1.27 (0.83–1.91)	0.262
TNM stage											
1	8	3	4	1				0.0483	0.4347		
2	39	9	22	8						3.62 (0.64–22.50)	0.152
3	24	8	13	3						1.55 (0.26–9.92)	0.636
4	29	8	8	13						3.52 (0.59–23.09)	0.175

BMI, body mass index; BMI = weight/height^2^. aCCI, age-adjusted Charlson Comorbidity Index. C3, Complement 3.

TNM stage, tumor node metastasis classification stage. OR, odds ratios. FDR, false discovery rate. Bold values indicate statistical significance after FDR correction or in the multivariable model (*P* < 0.05).

**Figure 4:**
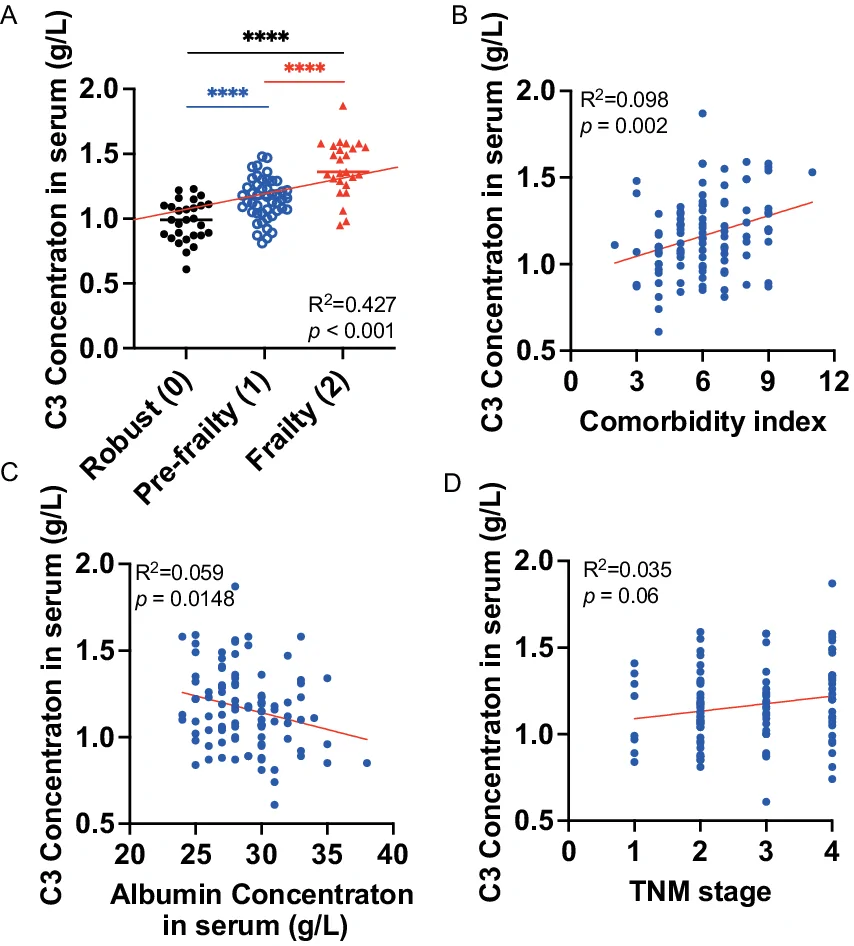
Validation in the CRC cohort identifies C3 as a key mediator of cancer-driven frailty. **(A)** Blood C3 concentration was positively correlated with frailty. **(B)** Blood C3 concentration was positively correlated with comorbidity index in patients with CRC (the comorbidity index is conceptually similar to the frailty index). **(C)** Blood C3 concentration was negatively correlated with serum albumin levels in patients with CRC. **(D)** Blood C3 concentration was not significantly associated with tumor stage in patients with CRC. CRC, colorectal cancer; TNM, Tumor-Node-Metastasis classification.

## Discussion

Patients with cancer frequently exhibit clinical manifestations that closely resemble those of frailty in older adults, including weight loss, reduced muscle mass and function, and fatigue. Consequently, considering cancer as an accelerated frailty model to identify key drivers and elucidate the underlying mechanisms of frailty represents a potential new research strategy. In the present study, we applied a phenome-wide MR (PheWAS-MR) framework to systematically investigate the causal relationships among 264 cancer-related diseases, 4,907 circulating blood proteins, and frailty. We identified direct causal associations between several common cancers and frailty and demonstrated that their underlying mediating mechanisms exhibit both shared and cancer-specific characteristics.

Frailty is a common phenotype in multiple cancer types. However, in our analysis, only approximately 15% of cancers exhibited a direct causal relationship with frailty (Figure 1C), which is substantially lower than the prevalence observed in clinical practice. One possible explanation is that multiple complex mechanisms drive cancer-induced frailty. Beyond the direct effects of circulating proteins, metabolic dysregulation, appetite control, energy homeostasis, and other biological processes may also substantially contribute to frailty development. These findings suggest that indirect regulatory pathways may play a major role. Furthermore, frailty is a highly complex phenotype influenced by numerous biological factors and is characterized by extensive pleiotropy. Because MR emphasizes direct causal inference and excludes pleiotropic genetic variants, this approach may inevitably generate a relatively high false-negative rate.

Cancer and frailty share several hallmark phenotypes, including weight loss, fatigue, reduced muscle mass, and impaired muscle function, suggesting the existence of common underlying biological mechanisms. GO enrichment analysis of the key mediating proteins identified in 5 cancer types revealed 20 shared molecular functions (Figure 3B), with pathways related to cell death and immune responses being the most prominently enriched (Figure 3C). A recent single-cell transcriptomic study by Xie et al. (31) demonstrated marked mast cell activation within the human CRC microenvironment. Notably, Li et al. (7) reported that mast cell activation may drive the transition to frailty during infectious conditions. Although our GO analysis did not directly enrich mast cell activation, these findings collectively suggest that cancer and frailty may share common immune-related regulatory mechanisms.

Additionally, previous studies have established that the tumor microenvironment is characterized by chronic inflammation enriched with multiple pro-inflammatory cytokines, including IL-1β, IL-6, and TNF-α. Yu et al. (6) further demonstrated that aging and frailty have distinct inflammatory niches. These findings suggest that cancer and frailty partially overlap in inflammatory niches, particularly through perturbations in TNF-mediated inflammatory signaling. Notably, extracellular vesicle-related GO terms were consistently enriched across all five cancer types (Figure 3B). This suggests that different cancers may directly influence key frailty-associated target organs, such as skeletal muscles, through extracellular vesicle-mediated intercellular communication.

At the mediator level, LCN2, F5, and C3 were identified in two of the five cancer types (Figure 3G), suggesting that these proteins may be key mediators shared across cancer types, linking cancer to frailty. Mosialou et al. (32) demonstrated that hypothalamic LCN2 regulates appetite through the melanocortin-4 receptor (MC4R). Subsequent studies by Lemecha et al. (33), Huisman et al. (34), and Olson et al. (28) extended these findings to cancer, demonstrating that hypothalamic LCN2 modulates appetite through MC4R in tumor-bearing models, thereby influencing cancer cachexia progression, and that targeting LCN2 attenuates cancer cachexia-associated muscle loss. Additionally, *in vitro* experiments conducted by Li et al. (29) demonstrated that F9, F10, and F13B, members of the same coagulation factor family as F5, promote senescence in human aortic endothelial cells. Since vascular aging is considered a major driver of systemic aging and frailty (35), these observations are highly relevant. Collectively, among the three high-frequency mediators identified using cancer as an accelerated frailty model, two have been experimentally implicated in frailty-related biological processes. These findings not only support their role as shared mechanisms underlying cancer-induced frailty but also highlight the efficiency of this screening strategy in identifying novel frailty mechanisms.

Notably, the number of key mediator proteins linking different cancer types to frailty varied substantially across cancer types. For example, kidney cancer yielded as many as 29 key mediators, whereas hemangioma and several other cancer types were associated with only 1–10 mediators (Figure 2C). These findings indicate that, beyond shared mechanisms, distinct cancer types may influence frailty through cancer-specific molecular pathways. Such heterogeneity was further reflected in the shared KEGG pathways between individual cancers and frailty. Kidney cancer exhibited enrichment for a considerably larger number of shared KEGG pathways, whereas breast cancer and hemangioma were enriched for only a single KEGG pathway. These observations suggest that pathological alterations in different organs contribute to frailty through distinct biological mechanisms, supporting the concept that aging progresses heterogeneously across organs (36). Notably, although the vascular system is among the earliest organs to age and cardiovascular diseases are established risk factors for frailty (37, 38), vascular tumors shared few molecular mechanisms with frailty, exhibiting highly distinct biological characteristics (Figure 2C). This suggests that systemic vascular disorders and localized vascular lesions, such as hemangiomas, influence whole-body aging and frailty through fundamentally different biological pathways and mechanisms.

Cardoso et al. (39) previously proposed C3 as a potential biomarker of frailty due to its established involvement in multiple neurodegenerative diseases; however, direct evidence linking C3 to frailty remains limited. Using clinical data from patients with CRC, we demonstrated a positive association between circulating C3 levels and frailty (Figure 4A). Furthermore, the Charlson Comorbidity Index, which underpins the frailty index approach for frailty assessment, was also positively associated with circulating C3 concentrations (Figure 4B). Conversely, albumin, a key indicator of malnutrition in frail individuals, was negatively associated with C3 (Figure 4C). These independent lines of evidence further strengthen the conclusion that C3 is involved in frailty. Importantly, C3 concentrations were not associated with tumor stage in our validation cohort (Figure 4D), suggesting that the marked heterogeneity of patients with advanced malignancies did not substantially confound our findings.

Muscle mass and muscle function represent two defining phenotypic characteristics of frailty. Consequently, investigating the role of complement component C3 in skeletal muscle may provide important mechanistic insights into its contribution to frailty development. In neuromuscular disorders, C3 contributes to myasthenia gravis pathogenesis through complement activation at the neuromuscular junction and has emerged as a promising therapeutic target (40). Beyond its role in neuromuscular diseases, C3 is also a key mediator of inflammatory responses. Gu et al. (41) demonstrated in C2C12 myoblasts and mouse models that bisphenol A activates the complement C3 cascade in muscle, leading to muscle atrophy and chronic muscle weakness through inflammation-dependent mechanisms. Conversely, Rouaud et al. (42) reported that C3 promotes myogenic progenitor cell differentiation *in vitro*, with intramuscular adipocytes identified as a major C3 source in the muscle microenvironment. These findings suggest that C3 exerts context-dependent regenerative and pathological effects in skeletal muscle. Consistent with this complexity, Nakamura et al. (43) reported that lower circulating C3 levels were associated with increased sarcopenia risk and poorer muscle function in community-dwelling older adults.

Beyond its effects on skeletal muscles, accumulating evidence suggests that C3 may contribute to frailty by regulating systemic energy metabolism. Previous studies have demonstrated that hypothalamic C3 participates in energy homeostasis by modulating food intake and energy expenditure (44, 45). Notably, Magliarelli et al. (46) reported that hepatic C3 expression is highly responsive to nutritional status. In a fasting-refeeding mouse model, hepatic C3 synthesis and secretion increased markedly during refeeding, indicating that C3 participates in nutrient sensing and systemic energy homeostasis. Collectively, these findings indicate that C3 may influence frailty through multiple interconnected biological pathways, involving hepatic nutrient sensing, hypothalamic appetite regulation, muscle inflammatory responses, neuromuscular junction integrity, and myogenic stem/progenitor cell differentiation. Nevertheless, the relative contributions of each pathway and the molecular mechanisms linking C3 to frailty remain largely undefined, requiring further investigation.

Frailty is one of the most prevalent geriatric syndromes; however, progress in understanding its pathogenesis and identifying effective therapeutic targets has been limited by the lack of robust cellular systems and efficient animal models. A translational strategy that leverages clinical comorbidity patterns to guide mechanistic research may offer a more efficient framework for studying frailty biology. Cancer and frailty share numerous clinical and biological characteristics, suggesting that cancer-associated pathological processes may accelerate mechanisms that also drive frailty. Building on this rationale, our study systematically evaluated the causal relationship between cancer and frailty and proposed cancer as a novel accelerated model for investigating the mechanisms underlying frailty. Furthermore, our findings provide preliminary evidence that complement C3 is involved in frailty development and suggest that frailty associated with most cancer types is likely mediated predominantly through indirect systemic mechanisms rather than by direct effects of the tumor itself. Together, our findings provide a new conceptual framework for investigating the biological basis of frailty and developing targeted therapeutic strategies.

Several limitations should be acknowledged. First, the GWAS data were derived from individuals of European ancestry, whereas clinical validation was performed in an East Asian population, warranting further validation across diverse ancestral populations. Second, although Mendelian randomization (MR) reduces confounding and strengthens causal inference, residual horizontal pleiotropy cannot be entirely excluded. Third, despite previous evidence of increased hepatic C3 levels in mice with C26 colorectal cancer-induced frailty, we measured C3 only in the human peripheral circulation and lacked tissue-level complement profiling; thus, the origin of circulating C3 remains uncertain. Fourth, the cross-sectional design precluded assessment of the temporal relationship between C3 elevation and frailty onset, highlighting the need for longitudinal studies. Fifth, potential confounders, including dietary intake, anticancer drug use, and baseline nutritional status, were not fully accounted for in the clinical analyses. Finally, clinical validation was restricted to colorectal cancer, and whether C3 represents a generalizable target for cancer-associated frailty across different malignancies requires further investigation.

## Data Availability

The original contributions presented in the study are included in the article/Supplementary material, further inquiries can be directed to the corresponding authors.

## Footnotes

1
https://r12.finngen.fi

2
http://www.R-project.org

3
http://sangerbox.com
